# How Media Use Influences the Fertility Intentions Among Chinese Women of Reproductive Age: A Perspective of Social Trust

**DOI:** 10.3389/fpubh.2022.882009

**Published:** 2022-05-10

**Authors:** Chuanlin Ning, Jing Wu, Yijie Ye, Nan Yang, Huacheng Pei, Hao Gao

**Affiliations:** ^1^School of Media and Communication, Shanghai Jiao Tong University, Shanghai, China; ^2^Faculty of Social Sciences, University of Ljubljana, Ljubljana, Slovenia; ^3^School of Economics and Finance, Shanghai International Studies University, Shanghai, China; ^4^School of Journalism and Communication, Nanjing Normal University, Nanjing, China

**Keywords:** media use, fertility intentions, social trust, Chinese reproductive-aged women, social media

## Abstract

**Background:**

The low fertility level has become a serious social problem in China. Previous research has argued the significant influence of media use and social trust on fertility intentions, but the interaction between the two variables and how they influence fertility intentions remain further investigation. This study explored the influence mechanism of media use on Chinese women's fertility intentions from the perspective of social trust.

**Methods:**

This study collected data from the 2017 China General Social Survey, investigated the relationships between variables through bivariate correlation coefficients, and explored the differences in fertility intentions among women of reproductive age (20–49). Also, this paper examined the influence of media use and social trust by regression analysis and tested the mediating role of social trust between media use and fertility intentions with Bootstrap sampling.

**Results:**

Women with different media use preferences, education levels, and family incomes have significant differences (*p* < 0.01) in fertility intentions. New media use negatively influences women's fertility intentions, while traditional media use has no significant influence on women's fertility intentions. Social trust significantly influenced women's fertility intentions and partially mediated the impact of new media use on fertility intentions.

**Conclusion:**

Online communication influences fertility intentions among Chinese women of reproductive age. It tends to influence their social trust by amplifying negative social news, affecting their fertility intentions further. This paper suggests the importance of strengthening social trust and online agenda-setting to improve women's fertility intentions that strategic information communication can change their perceptions of social trust.

## Introduction

Fertility is the driving force behind human development, and low fertility has become an important global issue, impacting the global society, economy, finance, and national security (1). The fertility rates in China have declined dramatically over the past few decades, and the annual total fertility rate only reached 1.65% from 2006 to 2019 (2). The low level of fertility caused a decreasing birth rate year by year. Mainland China's birth rate dropped to a record low of 7.52‰ in 2021 (3), which poses a huge challenge to China's demographic structure and social development (4). With the development of the social economy, the low fertility rate is no longer primarily driven by fertility policies but rather reflects the low fertility intentions in the current socio-economic context (5). In 2001, Bongaarts (6) proposed a model to explain the relationship between fertility intentions and fertility levels. Many studies have shown the correlation between fertility intentions and fertility rates from multiple perspectives (7–10). Among the factors influencing fertility rates, scholars identified low fertility intentions as an important cause of low fertility rates (11). They believed that fertility intentions could predict fertility rates, and the result is more accurate in the short term (12).

Studies have examined that media information is an important factor influencing fertility intentions. In addition to providing fertility-related health knowledge and policy propaganda to the public (13, 14), the media information can influence fertility intentions through moderating or mediating variables such as social role perceptions and gender attitudes (15, 16). In the mediated society constructed by traditional and new media, people gain diverse types of information, and new media platforms provide space for people to read, discuss and express (17). Topics like fertility policies, marriage and fertility values, which were rarely mentioned in the traditional media era, can be discussed on social media.

Social trust refers to an individual's belief about the general trustworthiness of others, and it is a part of an individual's worldview (18). Social trust plays a mediating role and thus influences people's certain behaviors in the complex-mediated environment (19). Studies have also examined that social trust is a significant factor in improving fertility intentions (20), thus influencing people's fertility behaviors. The influence mechanism needs further explanation in the mediated environment. In detail, whether media use can affect fertility intentions through social trust. If so, what role social trust plays in the mechanism.

Within the traditional Chinese culture, women were disadvantaged in their fertility choices, and childbirth is always necessary for common myths (21). Due to the patriarchal ideology, some women would not stop childbirth until they have a boy under the family and social pressures (22). Studies described women's fertility intentions under traditional Chinese fertility culture as ‘passive following', which is full of compromise (23). As society has progressed, women have access to higher education and earn higher social status. Chinese women have also gained more social roles and gradually broken free from the shackles that bind them to fertility (24). With the change in women's roles and attitudes in modern fertility discourse, women's fertility intentions deserve further attention.

On this basis, this study collected data from the 2017 Chinese General Social Survey (CGSS 2017), targeting women aged 20 to 49, to explore the influence of media use on fertility intentions among Chinese women and the role of social trust in the relationship between the two variables. Compared to previous research, the main contributions of this study are: (1) Scholars have explored the factors influencing women's fertility intentions from a socio-economic perspective. This study examined the influence of media on women's fertility intentions from the perspective of the mediated society. (2) Few studies based on the media perspective focused on Internet use, neglecting that the media environment is constructed with traditional and new media. The 'media use' in this study contains traditional media use, enhancing the understanding of the influence of diverse media types on women's fertility intentions. (3) Liu's research (16) selected gender attitudes as a mediating variable to investigate the influence of Internet use frequency on women's fertility intentions. Different from their views, this study considered social trust as an important factor in forming and influencing women's fertility intentions and expanded the knowledge of the social trust mechanism in media research.

## Literature Review

### Fertility Intentions of Women and the Influencing Factors

As a part of fertility decisions, fertility intention is a key indicator of measuring fertility (25). Fertility intentions can be defined from fertility desires, attitudes, and behaviors (26). Regarding fertility desires, fertility intentions include the desired number of children, the desired gender of children, and the interpregnancy interval (27, 28). Biologically, women are the ultimate bearers of childbirth. Women's fertility intentions decline more than men's intentions to have children as they get aging (29), which further directly affects fertility behaviors and thus fertility rates. Furthermore, scholars argued that female empowerment is an important reason causing the decline in female fertility intentions (30). Individualism, feminism, gender equality, and changes in marriage and family values also contributed to low fertility rates (31). Thus, women's fertility intentions become essential in a low-fertility environment. Regarding the CGSS data, this paper conceptualized fertility intentions as the desired number of children among reproductive-aged women.

The factors influencing women's fertility intentions are mainly related to the macro socio-economic and micro individual characteristics (28). In China, the role of individual fertility intentions in determining their fertility behaviors has become increasingly prominent with the gradual relaxation of fertility policies (32, 33). Many studies have examined the social status of women (34), gender role attitudes (16), education levels (35), family income (36), housing situation (37), cultural beliefs (38), and social security system (39) have significant influences on fertility intentions of women.

Combined with the relevant variables from the previous studies and the CGSS, this study determined education levels, family income, housing situation, and social status as control variables.

### Media Use and the Fertility Intentions of Women

In addition to traditional social, family, and individual factors, media as an important part of the social system has driven researchers' attention to exploring its impact on women's fertility. In terms of traditional media use, an Indian study showed that the official monopoly media, DoorDarshan India, significantly reduced women's fertility intentions through strong discussion about family planning and contraceptive use (14). In Indonesia, the expansion of private broadcast television and the growth of its subscribers have caused a low fertility rate as private television reinforced the promotion of modern contraceptives (40). Another study argued that media more likely influence fertility intentions of women with long-term media exposure. These women are more inclined to use contraception to reduce fertility possibilities and control the family size (41). Information and communication technology has rapidly developed since the 1990s, and new media based on the internet has been a vital factor in social change and an important trans-formative force in reconfiguring individual behaviors. Researchers began to focus on the impact of new media on women's fertility intentions. A study on Chinese women's fertility intentions indicated that attention to news on new media negatively correlated with women's fertility intentions (16). Cheng (13) found that social networks play an important role in spreading knowledge about contraception, reducing women's fertility intentions. Adair et al. (42) shared a finding based on content analysis of tweets that most people had a negative attitude to parenting, and single people who released the relevant content also had low fertility intentions.

In this regard, we proposed the following hypotheses:

H1: Media use negatively correlates with fertility intentions among Chinese women.

H1-1: Chinese women of reproductive age have lower fertility intentions when having more frequent traditional use.

H1-2: Chinese women of reproductive age have lower fertility intentions when having more frequent new media use.

### Social Trust, Media Use, and the Fertility Intention of Women

Studies on the impact of media use on women's fertility intentions found that media act in conjunction with other variables to influence fertility intentions. An experimental study from the United States indicated that media increased the willingness of non-student, unmarried, childless women to have children by reinforcing the portrayal of women's social roles (43). Liu et al. (16) believed that Chinese women have lower identification with Chinese traditional gender roles as the increase of their internet use, which further reduces their fertility intentions. Billari et al. (44) revealed a finding from German panel data that the internet can effectively mitigate work-family conflict by increasing the likelihood of remote working, which positively influences women's fertility intentions. Another research argued that social support from Facebook could reduce the abortion rate and thus increase fertility rates (45). The above studies have proven that media use can change the social roles of women and the perceptions of traditional gender roles and provide social support for women, further influencing the fertility intentions of women. The studies from different perspectives all emphasized the correlation between social factors and women's fertility intentions and the influence of media use on social factors.

Empirical research found that trust affects attitudes (46, 47) and causes individual and social behavior outcomes (48). Some scholars used social trust as a variable to explore its influence on fertility intentions and illustrated that trust is more important for fertility in countries with high trust and women with high education levels (49). A high-trust society is helpful for work-family balance, mainly achieved by the stability of key institutions and generous welfare support, thereby increasing fertility intentions (50). A study used panel data from 24 OECD countries during 1980–2004 and found that enhanced social trust can increase fertility rates (20). Thus, in a mediated society, whether media communication can influence social trust and further affect people's attitudes and intentions toward fertility desires a further exploration.

Early research on mass media and social trust suggested that mass media use has a certain impact on social trust. For example, a study with 1996 United States elections data indicated that the use of newspapers and television affects social trust (51). Another penal data research illustrated that mass media use affects social trust next year (52). With the advent of social media based on internet technology, scholars have found that social media influences social trust, but their findings are different. Some of them believed that social media use has more controllability of information that people can weed out mistrustful relations early and reduce the uncertainty (53). Also, local social media use can enhance interpersonal trust (54). A random sampling survey on US residents showed that people cultivate and build trusted relations through online interactions (55). Another survey on university students in Texas, USA, revealed a positive correlation between the intensity of Facebook use and social trust among university students (56). In contrast, Putnam (48) argued that the role of the internet in building trust is time-consuming, thus preventing face-to-face communication and limiting interaction with familiar people. Rheingold (57) also supported that fake identity online may lure vulnerable individuals into harmful relationships. In addition, researchers argued that the use of different media or different media use behaviors also has distinctions. Reading newspapers and watching entertainment content from television can enhance social trust while watching television news will weaken social trust (51). The internet positively impacts social trust when people use it for information exchange. Still, the internet negatively correlates with social trust when using it for social entertainment (58).

**Figure 1:**
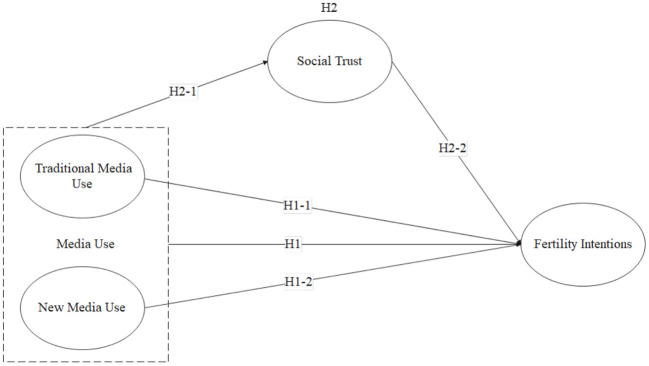
Hypothesized model.

Existing research has proved the influence of media use and social trust on fertility intentions and the impact of media use on social trust. However, the exact influence mechanisms are undetermined. Whether social trust plays a role in media use influencing fertility intentions needs further exploration. This study proposed the following hypotheses (see Figure 1 for full hypothesized model):

H2: Social trust mediates the relationship between media use and fertility intentions among women of reproductive age.

H2-1: Media use among women of reproductive age positively correlates with social trust.

H2-2: Social trust among women of reproductive age positively correlates with fertility intentions.

## Methods

### Study Design

This study explored the influence of media use and social trust on women's fertility intentions. First, we conducted descriptive statistics of media use, social trust, and fertility intentions of reproductive-aged women. Then, we used ANOVA to test whether there was a difference in fertility intentions of reproductive-aged women under different demographic variables. Furthermore, we utilized bivariate correlation coefficient and linear regression analysis to test the influencing relationships between the variables. Finally, we explored the influence of social trust on fertility intentions through a mediating effect model.

### Data Sources

CGSS is one of the authoritative academic survey projects in China and is widely used in Chinese social studies (59). National Survey Research Center at Renmin University of China (NSRC) is responsible for conducting the project, selecting samples through stratified random sampling and stratified-stage sampling. Participants in the projects are residents from 31 provinces, autonomous regions, and municipalities directly under the Central Government, excluding the Hong Kong Special Administrative Region, the Macau Special Administrative Region, and Taiwan. The latest CGSS data is updated to 2017 and includes a valid nationally representative sample of 14,670. Although the family planning regulations of China explain that the reproductive age of women is 15–49 (60), considering the legal age of marriage for women in China is 20 years old, this study selected data related to women aged 20–49. After deleting data with missing values and outliers, we obtained a valid sample of 2,649. Table 1 shows the sociodemographic information of selected samples.

**Table 1:** Sociodemographic information of the samples.

**Variable**	***n* (%) or Mean ±SD**
Age (year)	36.493 ± 8.657
**Education level**	
Uneducated	152 (5.74)
Primary school	466 (17.59)
Middle school	743 (28.05)
High school/technical secondary school	467 (17.63)
Junior college	326 (12.31)
Bachelor's degree	429 (16.19)
Master's degree and above	66 (2.49)
**Housing situation**	
Owner-occupied	801 (30.2)
Non-owner-occupied	1,848 (69.8)
Fertility intentions	1.851 ± 0.732
Family income	97,500.178 ± 146,573.099
Social trust	3.328 ± 1.043
Social status	4.311 ± 1.623
Traditional media use	2.148 ± 0.632
New media use	2.897 ± 1.104

### Variables Selection

#### Dependent Variable

##### Fertility Intentions

For fertility intentions studies, the American scholar George Gallup (61) introduced the concept of ideal family size for the first time. He used the question “what do you think is the ideal number of children for the average American family?” for the measurement, which has been widely used to measure people's intentions to have children since then (61). The question contained in CGSS2017, “how many children would you like to have if there were no policy restrictions” is consistent with the previous survey question measuring fertility intentions. Furthermore, scholars also used the same question to investigate fertility intentions (16). Thus, this study selected the data of this survey question to examine fertility intentions. Zero means that the respondent has no intentions to have children, and the higher value represents higher fertility intentions. Also, we standardized the data on fertility intentions of a 5-point Likert scale to maintain statistical consistency.

#### Independent Variable

##### Media Use

Media use is defined as “the extent to which an audience is exposed to a particular message or media content” (62). This study divided media use into traditional media use (newspapers, magazines, radio, television) and new media use (Internet and customized mobile news). CGSS asked the participants to answer their media use in the past year, measuring a Likert scale from 1 (never) to 5 (always). Besides, this study added the variable of media preference to compare the differences between traditional and new media use. We coded the situation that new media use is more than traditional media use as 1, representing the new media preference; new media use is less than traditional media use as 2, representing the traditional media preference; new media use equals traditional media use as 3, representing no media preference.

##### Social Trust

Social trust refers to shared expectations of each other, usually expressed as beliefs; that is, people behave wisely and mutually beneficial, when necessary, in their interactions with others. The shared expectations generate strong and stable relationships between people (63). This study chose the question, “Generally, do you agree that most people in the society can be trusted?” (64), as the indicator of social trust, responses ranging from 1 (strongly disagree) to 5 (strongly agree) with a five-point Likert scale.

##### Control Variables

The literature shows that education levels, family incomes, housing situation, and social status can influence women's fertility intentions, so this study viewed them as control variables. Specifically, the education level was selected from the question “What is your highest education?”; family income was assessed from annual household income, and the selected question is “What was your annual household income in 2016?”; housing situation was derived from the item “Do you own (including jointly with others) any property currently?”; social status refers to the question “In general, which social level are you at?”, ranging from 1 (the lowest level) to 10 (the highest level). This study coded the relevant variables to ensure statistical consistency: education levels (without education = 0 to postgraduate and above = 6), housing situation (owner-occupied = 1, non-owner-occupied = 0), family income was measured into different household income levels based on the average annual household income (low family income = 1, high family income = 2); social status was divided into three groups (values 1–3 were defined as low social status = 1, values 4–7 were middle social status = 2, and values 8–10 were high social status = 3).

## Results

### Descriptive Statistical Analysis

Table 2 provides a statistical description of media use, social trust, and fertility intentions of reproductive-aged women. In terms of media use, the frequency of new media use (M = 2.897) is higher than traditional media use (M = 2.148). Besides, social trust is relatively high (M = 3.328). The overall fertility intentions of reproductive-aged women are low (M = 1.851).

**Table 2:** Descriptive analysis.

**Variables**	**Mean**	**Standard deviation**
Traditional media use	2.148	0.632
New media use	2.897	1.104
Social trust	3.328	1.043
Fertility intentions	1.851	0.732

Table 3 compares the differences in fertility intentions of reproductive-aged women from the perspectives of media use preference, education levels, annual family income, social status, and social trust. The variance results show that women with different media use preferences, education levels, and family incomes have significant differences (*p* < 0.01) in fertility intentions. However, there are no significant differences in fertility intentions between reproductive-aged women with different housing situations, social status, and social trust.

**Table 3:** Differences in fertility intentions of reproductive-aged women from the different variables.

**Variables**	**Groups**	**Fertility intentions (M±SD)**	**F**	***p***
Education levels	Low (*n* = 1,828)	1.90 ± 0.72	23.968	0.000**
	High (*n* = 821)	1.75 ± 0.74		
Family income	Low (*n* = 1,761)	1.89 ± 0.72	17.401	0.000**
	High (*n* = 888)	1.77 ± 0.74		
Media use preference	New media (*n* = 1,895)	1.82 ± 0.75	8.591	0.000**
	Traditional media (*n* = 571)	1.96 ± 0.69		
Housing situation	No preference (*n* = 183)	1.83 ± 0.59	1.639	0.201
	Non-owner-occupied (*n* = 1,848)	1.84 ± 0.70		
	Owner-occupied (*n* = 801)	1.88 ± 0.80		
Social status	Low (*n* = 767)	1.80 ± 0.71	2.679	0.069
	Middle (*n* = 1,823)	1.87 ± 0.73		
	High (*n* = 59)	1.93 ± 0.85		
Social trust	Low (*n* = 724)	1.82 ± 0.77	1.803	0.165
	Middle (*n* = 365)	1.81 ± 0.72		
	High (*n* = 1,560)	1.87 ± 0.72		

**p < 0.01.

### Preliminary Analyses

Table 4 shows the correlations coefficients of the variables in this study, and social status (*p* < 0.05) and social trust (*p* < 0.05) are positively correlated with fertility intentions. Combined with the ANOVA results, women with higher social trust have the most significant fertility intentions (M = 1.87 ± 0.72). Education levels (*p* < 0.01), family income (*p* < 0.01), new media use (*p* < 0.01) are negatively correlated with fertility intentions. The results show that groups with high education level (M = 1.75 ± 0.74), high annual family income (M = 1.77 ± 0.74), and new media preference (M = 1.82 ± 0.75) have lower fertility intentions.

**Table 4:** Bivariate correlation between the variables.

	**1**	**2**	**3**	**4**	**5**	**6**	**7**	**8**
1. Fertility intentions	1							
2. Education levels	−0.140**	1						
3. Family income	−0.088**	0.526**	1					
4.Social status	0.046*	0.275**	0.353**	1				
5. Housing situation (owner-occupied)	0.025	−0.02	0.087**	0.066**	1			
6. Traditional media use	−0.03	0.341**	0.262**	0.198**	0.054**	1		
7. New media use	−0.107**	0.522**	0.408**	0.205**	−0.038*	0.350**	1	
8. Social trust	0.040*	0.075**	0.035	0.076**	0.01	0.044*	0.008	1

*p < 0.05. **p < 0.01.

This study conducted a hierarchical multiple regression analysis to investigate further the factors influencing women's fertility intentions. First, we used education levels, housing situation, social status, and annual family income as control variables for regression analysis with fertility intentions, forming Model 1. Then, we added media (traditional and new media) use and social trust based on the control variables to regress with fertility intentions, forming Model 2. As shown in Table 5, Model 1 indicates that education levels and annual family income negatively influence fertility intentions of reproductive-aged women, and social status positively influences fertility intentions. Model 2, with adding key variables, presents that under the control variables of education levels and social status, preference for new media use negatively affects fertility intentions of reproductive-aged women (B = −0.033, *p* < 0.05), and social trust positively affect fertility intentions (B = 0.030, *p* < 0.05). Regarding the correlation between traditional media use and fertility intentions is not significant, Hypothesis 1 is partially supported. In detail, H1-1 is not supported, and H1-2 is supported. Besides, H2-2 is also supported.

**Table 5:** Results of hierarchical multiple regression analysis (*n* = 2,649).

	**Model 1**					**Model 2**				
	**B**	**SE**	**t**	**p**	**β**	**B**	**SE**	**t**	**p**	**β**
Constants	2.028**	0.080	25.26	0.000	-	1.930**	0.097	19.91	0.000	-
Education levels	−0.066**	0.011	−6.19	0.000	−0.14	−0.061**	0.012	−5.17	0.000	−0.13
Housing situation (owner-occupied)	0.033	0.031	1.063	0.288	0.021	0.027	0.031	0.864	0.388	0.017
Social status	0.046**	0.009	4.921	0.000	0.102	0.044**	0.009	4.739	0.000	0.098
Annual family income	−0.009*	0.004	−2.20	0.028	−0.05	−0.010	0.004	−1.82	0.069	−0.04
Traditional media use						0.025	0.024	1.026	0.305	0.022
New media use						−0.033*	0.015	−2.11	0.035	−0.05
Social trust						0.030*	0.014	2.221	0.026	0.043
R^2^	0.029					0.033				
Adjusted R^2^	0.028					0.031				
F value	F (4.2644) = 20.057, *p* = 0.000					F (7.2641) = 12.952, *p* = 0.000				
ΔR^2^	0.029					0.004				
ΔF value	F (4.2644) = 20.057, *p* = 0.000					F (3.2641) = 3.406, *p* = 0.017				

*Dependent variable: fertility intentions*.

*p < 0.05 ^**^ p < 0.01.

### Testing for Mediation Effect

This study used social trust as the mediating variable to further investigate the influence of media use and social trust on women's fertility intentions, exploring the possible influencing mechanism. This study employed the hierarchical regression analysis to test the mediating effect. First, we took education levels, housing situation, social status, and annual family income as the control variables and added media use (traditional and new media) on this basis to regress with fertility intentions, forming Model 3. Then, we added media (traditional and new media) use based on the four control variables to regress with social trust, forming Model 4. Model 2 (seen as Table 5), Model 3, and Model 4 (seen as Table 6) showed that new media is positively correlated with social media (*p* < 0.01), while traditional media use is not significantly correlated with social trust. Thus, H2-1 is partially supported.

**Table 6:** Results of mediating effect analysis (*n* = 2,649).

	**Model 1**					**Model 2**				
	**B**	**SE**	**t**	**p**	**β**	**B**	**SE**	**t**	**p**	**β**
Constant	2.024**	0.087	23.213	0	-	3.145**	0.126	25.055	0	-
Education levels	−0.059**	0.012	−5.029	0	−0.125	0.057**	0.017	3.351	0.001	0.084
Housing situation	0.027	0.031	0.876	0.381	0.017	0.013	0.044	0.299	0.765	0.006
Social status	0.046**	0.009	4.876	0	0.101	0.041**	0.013	3.062	0.002	0.064
Annual family income	−0.008	0.004	−1.848	0.065	−0.044	−0.005	0.006	−0.76	0.447	−0.018
Traditional media use	0.026	0.024	1.076	0.282	0.023	0.041	0.035	1.161	0.246	0.025
New media use	−0.034*	0.015	−2.204	0.028	−0.052	−0.047*	0.022	−2.112	0.035	−0.05
Dependent variable	Fertility intentions					Social trust				
R^2^	0.031					0.011				
Adjusted R^2^	0.029					0.009				
F	F (6.2642) = 14.267, *p* = 0.000					F (6.2642) = 5.015, *p* = 0.000				

*p < 0.05 **p < 0.01.

For the issue of small values in the Model 1-4, R^2^ is a descriptive variable in most situations but not a test variable (65). Besides, the significant *p-*value and cons value still can fit better explanatory variable relationships even with a relatively low R^2^ value (66). R^2^ is a less fundamental statistic than regression slope, and it may change in response to variance changes in the independent variables despite the absence of structural differences across the population (67).

This study utilized the product of the coefficients method to examine the results of mediating effects. The product of the coefficients method has been widely used for its statistical efficacy over the causal step method (68). The product of the coefficients method contains two types. One is the Sobel test based on a normal sampling distribution with mediating effects. The other is asymmetric confidence interval based on a non-normal sampling distribution with mediating effects, including Bootstrap and product distribution. A simulation study by Mackinnon et al. (69) found that Bootstrap had the highest statistical efficacy in mediating effects analysis. Thus, this study used Bootstrap sampling for testing significance. In this study, a denotes the regression coefficient of media use to social trust; b represents the regression coefficient of social trust to fertility intentions; c represents the regression coefficient of media use to fertility intentions (without mediator), which is the total effect; and c' represents the regression coefficient of media use to fertility intentions (with a mediator), which is the direct effect. According to the procedure, the bootstrap sampling test for mediating effect was strictly processed. If a and b are significant, and c' is significant, and a^*^b is synonymous with c,' it is partially mediated. Suppose at least one of a and b is not significant, and the 95% confidence interval (BootCI) for a^*^b includes 0. In that case, the mediating effect is insignificant (70).

**Figure 2:**
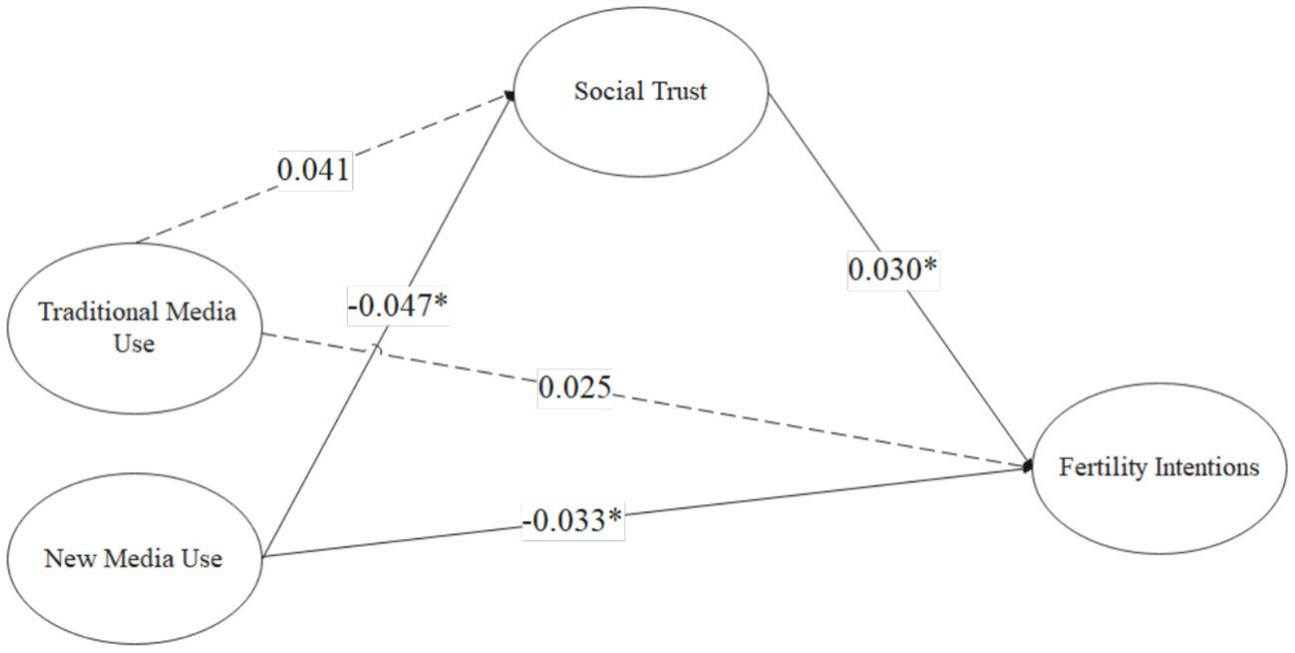
Final model.

Table 7 tells that a, b and c' are significant in Model 4, and a^*^b is synonymous with c', suggesting a mediating effect. Social media plays a mediating role in the relationship between new media use and fertility intentions. We combined the above findings and concluded that new media use negatively influences fertility intentions of reproductive-aged women and further influences fertility intentions through the mediating effect of social trust. Thus, H2 is partially supported. The final model is depicted in Figure 2.

**Table 7:** Results of mediating effects.

**Item**		**Model 3**	**Model 4**
c	Total effect	0.026	−0.034*
a		0.041	−0.047*
b		0.030*	0.030*
a*b	Mediating effect value	0.001	−0.001
a*b	(Boot SE)	0	0
a*b	(z value)	80.413	−63.99
a*b	(*p*-value)	0	0
a*b	(95% BootCI)	−0.001 ~ 0.003	−0.004 ~ 0.001
c'	Direct mediating effect	0.025	−0.033*
Test result		Not significant	Partially mediated
Effect account		0	4.14%

*p < 0.05.

*Model 3: traditional media use-social trust-fertility intentions*.

*Model 4: new media use-social trust-fertility intentions*.

## Discussion

### Issue of Low Fertility in Mainland China: Increase Women's Fertility Intentions

As mentioned above, the fertility rate in mainland China has dropped to a record low in 2021. Prolonged low fertility rates will cause rapid decline and high aging of the population, and a range of adverse socio-economic consequences, putting China at significant risk of fertility crisis (4). Demographers consider fertility intentions an important predictor of fertility rates (71), a determining factor of fertility behaviors, and a key factor influencing fertility rates (72). In terms of the correlation between fertility intentions and fertility behaviors, the prevailing view is that the actual fertility behaviors of people are lower than their reported fertility intentions in a low-fertility society at the end of the demographic transition (73). Wu and Li (4) summarized the results of the surveys on fertility intentions in China in recent years and found that the average ideal number of children for the reproductive-aged Chinese people was significantly lower than the replacement level. Besides, their average intended number of children and the desired number of children were lower than the ideal number. The emergence of be-low-replacement fertility intentions marks a new stage in which low-fertility countries are stepping (74). The findings from this study also suggested low fertility intentions among Chinese women of reproductive age, which explained the low fertility rates in mainland China.

The Chinese government has noticed the issue of low fertility rates. It has begun to relax its long-standing one-child policy, such as the selective two-child policy in 2013 and the universal two-child policy in 2015. The effect of these policies on increasing the births number was relatively weak (75). In June 2020, the Chinese government announced implementing the three-child policy after releasing the seventh national census results. Some local governments complemented support such as fertility subsidies, housing security, and childcare support (76). However, the birth rates in China still declined after implementing the new policy. Although the short duration of the policy can explain the decline, the low birth rates in 2021 also suggested re-examining the relationship between fertility intentions and fertility behaviors. The governments need to simultaneously increase fertility intentions and behaviors of the reproductive-aged population when im-proving the fertility policies and enhancing relevant support (77). Factors influencing fertility intentions involved individual characteristics and structural factors such as economic, social, cultural, and institutional factors, requiring adjusting of structural factors to construct a fertility-friendly society (4).

### Influence Mechanisms of Media Use on Women's Fertility Intentions

This study found that the influence of different media use on fertility intentions of reproductive-aged women was inconsistent. Groups with new media preference have significantly lower fertility intentions than groups with traditional media preference, and the increased new media use reduced fertility intentions of reproductive-aged women.

On the one hand, China's media system contributed to the above situation. Traditional media, such as radio, television, newspapers, and magazines, release reports under the premise of the party-controlled media and the state-owned system and take responsibility for propagating the policies and guidelines of the party and the nation (78). Thus, fertility issues in traditional media mainly focus on introducing and interpreting national fertility policies. On the other hand, traditional media communications are mostly one-way communication with weak interactions between media and audiences and between the audiences themselves.

In contrast, information dissemination on the internet is open and anonymous, allowing people to express their opinions of events and issues freely. With the interactions and discussions, people easily explore a certain issue deeply. In the context of traditional Chinese culture, the fertility issue has often been discussed within interpersonal communication, especially the transmission of fertility conceptions from parents to offspring. Research confirmed that traditional concepts about fertility, such as “a man should have a wife, and a woman should have a husband,” “carrying forward the family with a son,” and “raising children for the old,” are still transmitted intergenerationally in rural areas of China (79). People obtain fertility information from diverse recourses in the digital age and form more rational and pluralistic fertility conceptions through direct opinion expressions and interactions.

Furthermore, compared to traditional media, which promote fertility policies actively, online platforms disseminate fertility information and concepts via more diverse formats. First, new media platforms allow more information, including news reports, individual narratives, and expressions about fertility and parenting. Individual expressions on fertility issues can be positive or negative, while traditional media news is always positive. Studies showed that individual narratives are more persuasive to audiences than factual reports (80, 81). From the content of Chinese social media, many women expressed the pain of childbirth and parenting on social media, which is more likely to influence women's fertility intentions than positive fertility policy propaganda. Also, the online dissemination of controversial policies and negative marriage and fertility news easily triggered and fermented negative emotions on social media, which further influences the fertility intentions of reproductive-aged women. For example, the Supreme People's Court of Shandong Province posted a WeChat article titled “Divorce cannot be sought solely on the grounds of cheating” on January 3, 2022, which quickly fermented on Sina Weibo and then generated strong negative emotions. Many female netizens expressed as “stay safe without marriage and childbirth” and “stay away from men or be unfortunate,” reflecting women's general distrust of men and marriage. A big data analysis of public opinion on the three-child policy on Sina Weibo indicated that Weibo users were generally negative and neutral to the policy and supporting measures. The negative emotions mainly reflected the worries about implementing the supporting policies, the high cost of raising children, and the fragmentation of retirement, fertility, and education policies (82). The negative public opinion and emotions indicated the public distrust in current fertility policies and the social support system, thus expressing negative fertility intentions.

### How to Increase Fertility Intentions Through Improving Social Trust

This study suggested that social trust positively influences fertility intentions and mediating between media use and fertility intentions among reproductive-aged women. Many studies have confirmed the positive role of social trust in fertility intentions. The influence mechanism is that groups with high social trust are more willing to rely on social services and security institutions to support fertility, parenting, and education, helping women maintain the relationship between work and fertility (52). Improvement of the social systems of fertility, childcare, and education needs the support of trusted policies and supporting measures. In terms of enhancing social trust, scholars proposed to increase both interpersonal and institutional trust (83). Interpersonal trust needs moral and educational influences and the reduction of social polarization and income disparity (84). Besides, reducing social conflicts and ensuring public safety from the public policies are also necessary for enhancing interpersonal trust (85). To increase institutional trust, public policies should be responsible for the fair and effective implementation of the legal system, enhancement of social justice, insurance of employment, and economic development and equity in income and economic opportunities (83).

How to increase women's fertility intentions through social trust improvement is also a matter for media. The literature revealed that media has a certain impact on social trust (13, 14, 25, 42, 43). This study showed that new media use among Chinese reproductive-aged women positively correlated with social trust. Therefore, in the mediated society, there is a particular need to consider how social trust can be enhanced via new media platforms and further moderate women's fertility intentions. This study found that new media use negatively influenced the fertility intentions of reproductive-age women. Scholars argued that new media greatly increases women's perceptions of the potential fertility risks, such as family finances, children's education, marital status, childbirth risks, postpartum depression, postpartum recovery and life quality, which reduces women's fertility intentions (86). Women's perceived risks of fertility also reflected the distrust in fertility policies and social support systems. On this occasion, the government has to monitor online public opinion and sentiment and timely respond to women's concerns about fertility issues. Furthermore, the government can utilize the new media agenda-setting to guide people to face fertility rationally. For example, the government can provide a detailed introduction and interpretation of the fertility policies and social support system and conduct scientific communication on fertility. Some countries have already implemented their online fertility health promotion programs. The Australian government has funded interactive websites and social media accounts, gaining over 5 million annual visits to the websites and 96,000 users' engagement on social media (87). European countries have also set up websites on fertility topics, promoting public discourse around fertility issues and education and aiming to increase scientific awareness of fertility among their public (88).

## Conclusion

This study used CGSS data to examine that new media use negatively influences fertility intentions of reproductive-aged women, and social trust plays a mediating role between media use and fertility intentions. Besides, we further explored the mechanisms of how media use and social trust influence fertility intentions. This study provides a new perspective when researching fertility intentions and suggests strategies for the Chinese government to improve fertility intentions. The governments can utilize agenda-setting and change media reporting strategies.

However, this study has certain limitations and desires further qualitative research on this topic. On the one hand, this study used secondary data, and the research data is somewhat limited by the original survey questions. For example, the items of media use in the survey are limited. Our future research will further explore the relations between media use, social trust and women's fertility rates with a self-designed questionnaire. Also, future research will take the new fertility policies in China into account, such as the three-child policy. On the other hand, this study focused on women's fertility intentions, and the future study will expand the research attention to a more general dimension, which concludes the intentions of the male and the female.

## Data Availability Statement

Publicly available datasets were analyzed in this study. This data can be found at: http://cgss.ruc.edu.cn.

## Ethics Statement

The studies involving human participants were reviewed and approved by the Institutional Board at Nanjing Normal University. Written informed consent for this study was not required in accordance with local legislation and national guidelines. Written informed consent for participation was not required for this study in accordance with the national legislation and the institutional requirements.

## Author Contributions

CN and HG designed the study and revised the manuscript. YY and HP analyzed the data. NY and JW were involved in manuscript writing. All authors have read and approved the manuscript.

## Funding

This study was funded by the National Social Science Foundation of China (Grant No. 17CXW016).

## Conflict of Interest

The authors declare that the research was conducted in the absence of any commercial or financial relationships that could be construed as a potential conflict of interest.

## Publisher's Note

All claims expressed in this article are solely those of the authors and do not necessarily represent those of their affiliated organizations, or those of the publisher, the editors and the reviewers. Any product that may be evaluated in this article, or claim that may be made by its manufacturer, is not guaranteed or endorsed by the publisher.

## Abbreviations

CGSS: Chinese General Social Survey.
